# Epifluorescence Microscopy with Image Analysis as a Promising Method for Multispecies Biofilm Quantification

**DOI:** 10.4014/jmb.2209.09045

**Published:** 2023-01-18

**Authors:** Ji Won Lee, So-Yeon Jeong, Tae Gwan Kim

**Affiliations:** Department of Microbiology, Pusan National University, Pusan 46241, Republic of Korea

**Keywords:** Epifluorescence microscopy, image analysis, crystal violet staining, biofilm quantification, multispecies biofilm

## Abstract

Epifluorescence microscopy with image analysis was evaluated as a biofilm quantification method (*i.e.*, quantification of surface area colonized by biofilms), in comparison with crystal violet (CV) staining. We performed different experiments to generate multispecies biofilms with natural and artificial bacterial assemblages. First, four species were inoculated daily in 16 different sequences to form biofilms (surface colonization, 0.1%−56.6%). Second, a 9-species assemblage was allowed to form biofilms under 10 acylase treatment episodes (33.8%−55.6%). The two methods comparably measured the quantitative variation in biofilms, exhibiting a strong positive relationship (*R*^2^ ≥ 0.7). Moreover, the two methods exhibited similar levels of variation coefficients. Finally, six synthetic and two natural consortia were allowed to form biofilms for 14 days, and their temporal dynamics were monitored. The two methods were comparable in quantifying four biofilms colonizing ≥18.7% (*R*^2^ ≥ 0.64), but not for the other biofilms colonizing ≤ 3.7% (*R*^2^ ≤ 0.25). In addition, the two methods exhibited comparable coefficients of variation in the four biofilms. Microscopy and CV staining comparably measured the quantitative variation of biofilms, exhibiting a strongly positive relationship, although microscopy cannot appropriately quantify the biofilms below the threshold colonization. Microscopy with image analysis is a promising approach for easily and rapidly estimating absolute quantity of multispecies biofilms.

## Introduction

Biofilms have received extensive attention for the past several decades, because of their ecological, environmental, and pathological implications [1, 2]. They are defined as an aggregate of microbial cells embedded in a matrix of self-produced extracellular polymeric substances (EPS), mainly composed of polysaccharides, proteins, nucleic acids, and lipids, which are responsible for adhesion to surfaces and cohesion [3]. They have considerable biodiversity, spanning prokaryotic and eukaryotic microbial species [4, 5]. Microorganisms colonize surfaces and develop biofilms together with other organisms, resulting in a 3D expansion of the biofilm over time, which is influenced by various biological (*e.g.*, species traits and intra- and inter-species interactions) and non-biological (*e.g.*, shear stress, nutrients, temperature, and pH) factors [2, 6-8]. In general, biofilms vary greatly in structure (*i.e.*, architecture, texture, or biogeography) and composition (*i.e.*, composition of cells and EPS, EPS chemical composition, or species composition) [2, 3, 9, 10].

There are a wide variety of quantification methods available for multispecies biofilms, including gravimetric analysis, quantitative PCR (qPCR), metabolic activity assay, crystal violet (CV) staining, and microscopy [11, 12]. CV staining, one of the most commonly used methods, can help to estimate the total amount of cells and EPS in biofilms by quantifying the CV dye binding to them [13]. This technique has been widely used to quantify biofilm formation in microplates [14, 15]. qPCR is used as a DNA quantification method to quantify the number of cells present in biofilms, using primer sets targeting specific taxa [16]. An enzyme activity assay is used to estimate biofilm quantity by measuring the metabolic activity of cells in biofilms (*e.g.*, tetrazolium salt XTT assay) [14]. Microscopy (*e.g.*, epifluorescence microscopy and confocal laser scanning microscopy) is commonly used to observe the surface colonization and 3D structure of biofilms [17-19]. The concurrent use of microscopy and image analysis allows quantification of biofilms by measuring the area colonized by them [19, 20]. Moreover, confocal microscopy can be used to estimate biofilm volume by measuring its thickness and surface colonization [21, 22].

Epifluorescence microscopy with image analysis can be a promising quantification method for multispecies biofilms. Epifluorescence microscopes and image analysis programs are easy to employ, easily accessible, and inexpensive to use. An example is ImageJ software, an open-source image analysis program that can be applied easily and according to the user’s needs [23]. In addition, this technique can be used to measure the absolute quantity of biofilm colonization on a surface for a direct comparison of quantification results among biofilms, laboratories, and species. We hypothesized that the surface area colonized by a biofilm reflects the total amount of its formation. The main purpose of this study was to evaluate epifluorescence microscopy with image analysis for multispecies biofilm quantification, in comparison with CV staining. To induce variations in biofilm quantity, different multispecies biofilms were generated by varying species combinations, and a multispecies biofilm was modified by different acylase treatment episodes. In addition, eight consortia (activated sludge and soil consortia plus six synthetic consortia) were allowed to form biofilms, and their temporal biofilm dynamics were observed.

## Materials and Methods

### Microorganisms and Consortia

The twenty-four bacterial isolates used in this study are shown in Table 1. They originated from different environments, such as forest soil, wetland soil, mudflats, and activated sludge (AS) [24-26]. The isloates were maintained in R2A agar plates (MB Cell, KisanBio Co., Ltd., Korea) as pure colonies before use. Two bacterial consortia originating from soil (Yonghwasil wetland, Korea) and AS (a sewage treatment plant, Busan, Korea) were used in this study. In brief, 1 ml of soil or AS supernatant was transferred to a 250-ml flask containing 50 ml of low-strength R2A (LS-R2A) medium and incubated at 25°C with agitation at 200 rpm for 2 days [16]. LS-R2A medium contained 1 g/l R2A (MB cell) instead of 3.15 g/l recommended by the manufacturer. This low-strength medium was used to cultivate the microbial consortium and biofilms in this study. The consortia were obtained by subsequent transfer (10 times) to LS-R2A medium with cycloheximide (50 mg/l, Fujifilm Wako Pure Chemical Corp., Japan) to exclude eukaryotic organisms. The soil and AS consortia were stored at −70°C before use.

### Inoculum Preparation

Each pure bacterial colony was inoculated into a 250-ml flask containing 100 ml of LS-R2A medium. A suspension (100 μl) of soil or AS consortium was inoculated into a 500-ml flask containing 300 ml of LS-R2A medium. The cultures were incubated at 30°C with agitation at 200 rpm for up to 2 days. Cells were harvested by centrifugation at 3,000 ×*g* for 20 min and suspended in sterile 0.9% NaCl solution. For measuring dry cell weight, cell suspension was filtered through a glass fiber filter paper (GF5, CHMLAB Group, Barcelona, Spain) and then dried at 105°C for 2 h. All cell suspensions were diluted to a final concentration of 1 g/l with sterile 0.9% NaCl solution and stored at -70°C.

### Biofilms with Different Species Assembly Sequences

Sterilized 96-well plates (SPL Life Sciences Co., Ltd., Korea) with flat bottoms were used to form biofilms. The well dimensions were 6.9 mm and 10.8 mm for the diameter and height, respectively. A preliminary study was performed to explore the effects of adding sequences from four species (*Acinetobacter* sp. YS01, *Bacillus* sp. AS03, *Sphingopyxis* sp. NM1, and *Xanthomonas translucens*) on biofilm development. In brief, 4-species biofilms were constructed by adding a species daily for 4 days (4^4^, 256 combinations) and quantified using CV staining. In this study, a species was inoculated daily in 16 species-adding sequences exhibiting considerable variation (Table S1). One hundred microliters of cell suspension (1 g/l) was inoculated onto the bottom of each plate, which was then incubated for a day at 25°C with agitation at 40 rpm. The bacterial suspensions were carefully discarded by pipetting, and 100 μl of new cell suspension was added to the wells. They were incubated at 25°C with agitation at 40 rpm. This step was repeated twice. There were three replicates per sequence.

### Biofilms with Different Acylase Treatment Episodes

A consortium composed of *Acinetobacter* sp. YS01, *Bacillus* sp. AS03, *Escherichia coli*, *Enterobacter* sp. YS02, *Sphingobium xanthum*, *Sphingopyxis* sp. NM1, *Microbacterium* sp. NM2, *Staphylococcus warneri*, and *Xanthomonas translucens* was used as an inoculum. The different daily acylase treatment episodes are shown in Table S2. One hundred microliters of the consortium, with or without acylase (Sigma-Aldrich, USA; final concentration of 10 mg/l), was inoculated onto the bottom of a plate. The plate was incubated at 25°C with agitation at 40 rpm for 5 days, during which the suspension was replaced daily with the same volume of fresh LS-R2A medium with or without acylase (final concentration, 10 mg/l). There were three replicates per each treatment.

### Temporal Biofilm Variations

Eight consortia (soil and AS consortia plus six synthetic consortia composed of bacterial isolates) were used to study temporal biofilm dynamics. Six synthetic consortia (3-, 5-, 7-, 10-, 15-, and 20-species assemblages) were constructed by adding species in a random sequence (Table S3). A consortium (100 μl) was inoculated onto the bottom of a plate, which was incubated at 25°C with agitation at 40 rpm for 14 days. The suspension was replaced daily with an equal volume of fresh LS-R2A medium. Biofilms were quantified on days 3, 5, 7, 10, and 14. There were three replicates per consortium.

### Crystal Violet Staining

Biofilms were quantified using the CV staining method [27]. In brief, a plate was submerged in a 1-L beaker filled with 800 ml of distilled water to remove unattached cells and medium components before staining. Two hundred microliters of 0.1% CV staining solution (Merck KGaA, Germany) was added to each well of the plate and incubated at 25°C for 15 min. The plate was submerged and rinsed in a 1-L beaker filled with 800 ml of distilled water to remove the dye. The rinsed plate was turned upside down and air-dried for 24 h. For dissolving the CV, 200 μl of 30% acetic acid (Duksan Co., Ltd., Korea) was added to each well and incubated at 25°C for 15 min. After dissolution, 125 μl of the solution was transferred to a cuvette and filled with 1 ml of 30% acetic acid. Absorbance of solubilized CV was determined at 550 nm using a spectrophotometer (UV-1800, Shimadzu Corp., Japan).

### Microscopy with Image Analysis

Biofilms on bottom surfaces were quantified using epifluorescence microscopy with image analysis. Each plate was submerged in a 1-L beaker filled with 800 ml phosphate-buffered saline solution (PBS; pH 7.3) to remove unattached cells and medium components before staining. Two hundred microliters of 300 nM 4′,6-diamidino-2-phenylindole dihydrochloride (DAPI) (Roche Diagnostics GmbH., Germany) staining solution was added to each well of the plates and incubated at 25°C for 15 min. After staining, each plate was submerged and rinsed three times in a 1-L beaker filled with 800 ml PBS solution to remove excess dye. Biofilms on the bottom surfaces were observed at 200× magnification using an epifluorescence microscope (Carl Zeiss GmbH., Germany) equipped with an LD A-Plan 20×/0.35 Ph1 lens. DAPI fluorescence was detected using the filter set 90 high efficiency (HE) LED. Images from 10 random spots on the outskirts of each well bottom were captured using an Axiocam 506 color camera (Carl Zeiss) and ZEN 2.3 software (Carl Zeiss). Every micrograph with biofilms was saved and analyzed to estimate biofilm growth. To alleviate auto-fluorescence in the wells, DAPI staining was performed as aforementioned on empty wells at each sampling time. On each captured image, the area colonized by biofilm was quantified using the ImageJ version 1.8.0 software (https://imagej.nih.gov/ij). The autofluorescence level was set as a threshold for positive calls. Pixels brighter than the threshold level (representing biofilms) were counted and converted into area.

### Statistical Analysis

Linearity test (Y = *a·x+b*) was performed to determine the relationship between the measurements by epifluorescence microscopy with image analysis and CV staining. We performed linear regression using the Sigma-Plot software version 10 (Systat Software, USA).

## Results

### Biofilms with Different Species Assembly Sequences

**Fig. 1:**
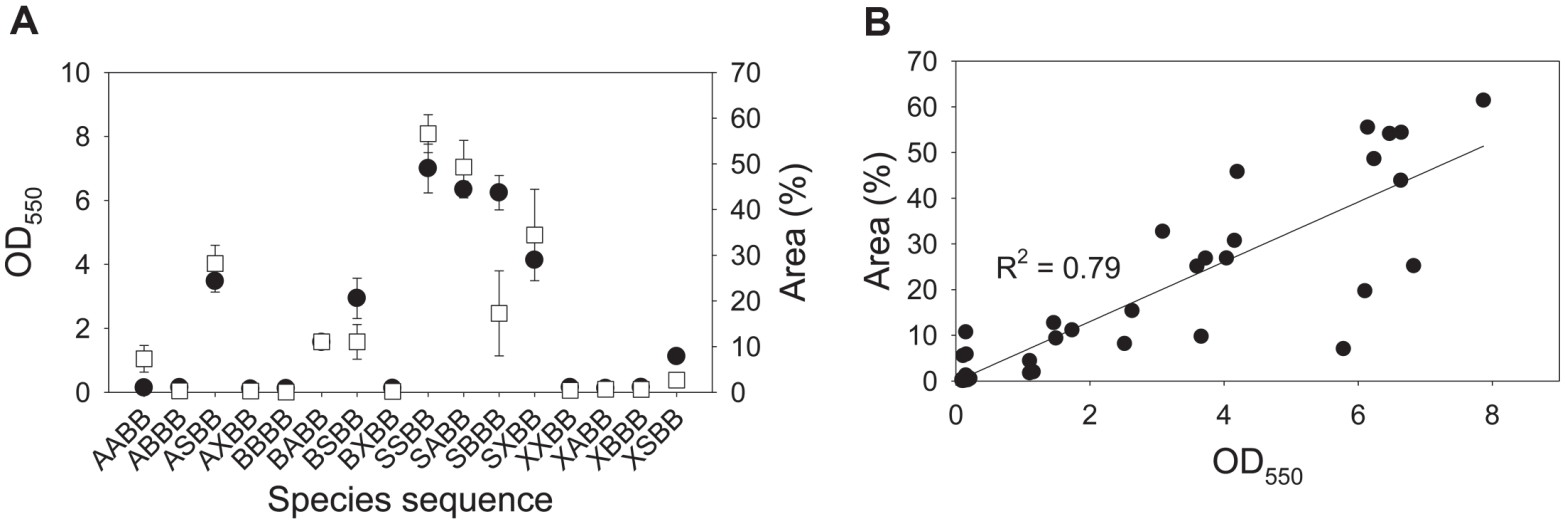
Biofilm variation by adding sequences of species. (**A**), biofilm quantification using epifluorescence microscopy with image analysis (□) and crystal violet staining (●); and (**B**), the linear relationship between their measurements. A, *Acinetobacter* sp. YS01; B, *Bacillus* sp. AS03; S, *Sphingopyxis* sp. NM1; and X, *Xanthomonas translucens*. Error bars represent standard deviation of the mean (*n* = 3).

Some biofilm micrographs obtained in this study using epifluorescence microscopy are shown in Figs. S1-S3. Multispecies biofilms were generated from 16 species assembly sequences (Fig. 1). Microscopy showed that SSBB colonized the largest area (56.60%), followed by SABB (49.30%), SXBB (34.43%), ASBB (28.19%), and SBBB (17.27%). Similarly, CV staining revealed that SSBB formed the largest biofilm (Optical density at 550 nm (OD) , 7.00), followed by SABB (6.34), SBBB (6.24), SXBB (4.13), and ASBB (3.47). The average colonized area was 13.79% (ranging from 0.05% to 56.60%), and the average OD (by CV staining) was 2.12 (ranging from 0.11 to 7.00)(Fig. 1A). A variation coefficient (*i.e.*, standard deviation divided by the mean) was computed to estimate the extent of variability in measurements by microscopy and CV staining [28]. The variation coefficients were 1.35 and 1.21 for microscopy and CV staining, respectively. The linearity test between the microscopic and CV staining estimates exhibited a strong positive relationship (*R*^2^ = 0.79) (Fig. 1B).

### Biofilms with Different Acylase Treatment Episodes

**Fig. 2:**
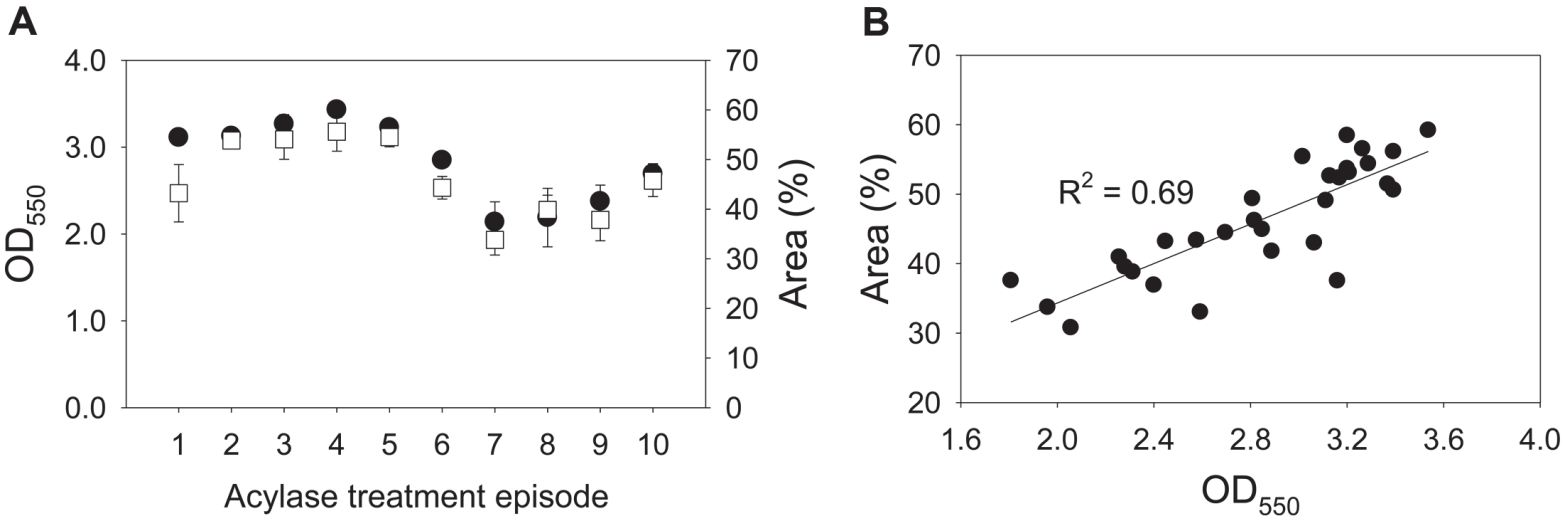
Biofilm variation with different acylase treatment episodes. (**A**), biofilm quantification using epifluorescence microscopy with image analysis (□) and crystal violet staining (●); and (**B**), the linear relationship between their measurements. Error bars represent standard deviation of the mean (*n* = 3).

A 9-species assemblage was allowed to form biofilms under 10 acylase treatment episodes (Fig. 2). The average colonized area was 46.28% (ranging from 33.82% to 55.60%), and the average OD was 2.84 (ranging from 2.14 to 3.43) (Fig. 2A). Their variation coefficients were nearly equal (0.17 vs. 0.17). A linearity test showed a strong positive relationship between the microscopic and CV staining measurements (*R*^2^ = 0.69) (Fig. 2B). A comparison of the treatment episodes revealed that acylase addition on day 1 was only effective in reducing biofilm development (OD, 3.23 vs. 2.45; and area, 52.25% vs. 40.32% for 1–5 biofilms with no acylase vs. 6–10 biofilms with acylase on day 1, respectively) (*p* < 0.05).

### Temporal Biofilm Variations

**Fig. 3:**
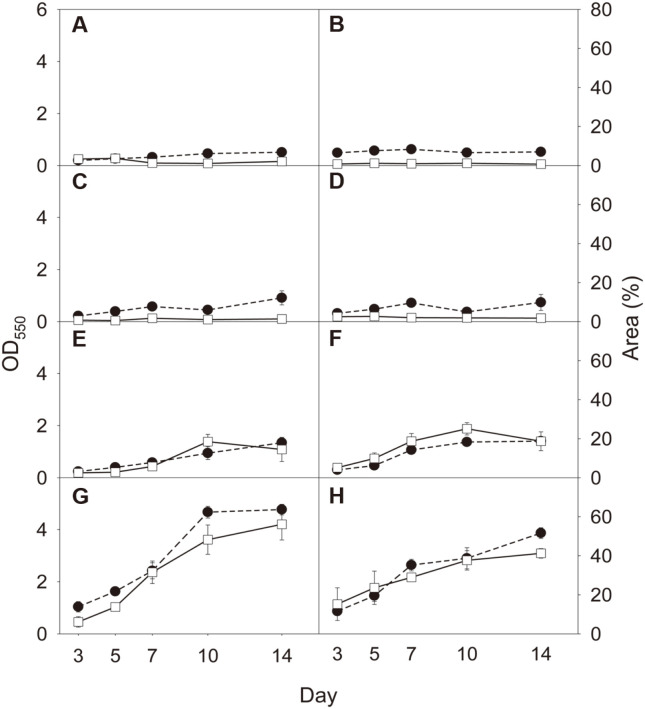
Temporal biofilm variation. Biofilms were quantified using epifluorescence microscopy with image analysis (□) and crystal violet staining (●). Error bars represent standard deviation of the mean (*n* = 3). (**A**), AS consortium; (**B**), soil consortium; (**C**), 3-species consortium; (**D**), 5-species consortium; (**E**), 7-species consortium; (**F**), 10-species consortium; (**G**), 15-species consortium; and (**H**), 20-species consortium.

**Fig. 4:**
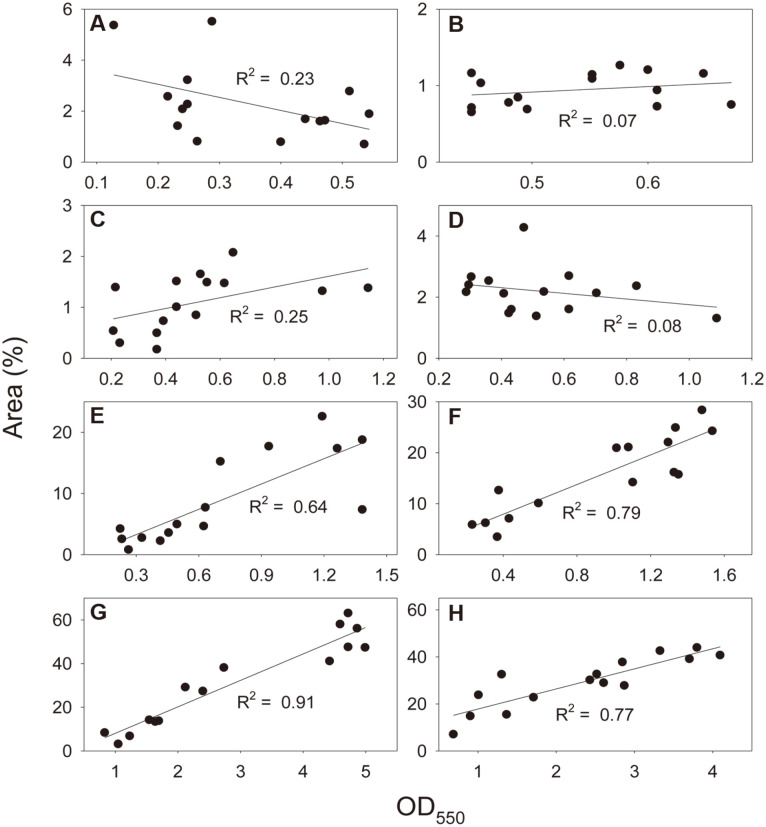
Linearity test results between the measurements by epifluorescence microscopy with image analysis and crystal violet staining (Fig. 3). (**A**) AS consortium; (**B**), soil consortium; (**C**), 3-species consortium; (**D**), 5-species consortium; (**E**), 7-species consortium; (**F**), 10-species consortium; (**G**), 15-species consortium; and (**H**), 20-species consortium.

Eight consortia (AS and soil consortia plus six synthetic consortia) were allowed to form biofilms for 14 days (Fig. 3). Using both methods, we observed differences among the eight biofilms and their temporal variations. Weak relationships were observed between the measurements by CV staining and microscopy in AS, soil, 3-species, and 5-species biofilms (*R*^2^ ≤ 0.25) (Figs. 4A-4D). In AS biofilm, CV staining showed a temporal increase in biofilm formation (linearity test, slope *p* < 0.05), whereas microscopy did not (*p* > 0.05) (Fig. 3A). The colonized area ranged from 1.06% to 3.67% (variation coefficient, 0.52), and OD ranged from 0.19 to 0.51 (0.38). In soil biofilm, neither method showed a temporal change in biofilm formation (*p* > 0.05) (Fig. 3B). The extent of colonization ranged from 0.71% to 1.15% (0.22), and the OD ranged from 0.49 to 0.62 (0.10). In 3-species biofilm, CV staining exhibited a temporal increase (*p* < 0.05), whereas microscopy did not (*p* > 0.05) (Fig. 3C). The extent of colonization ranged from 0.56% to 1.74% (0.44), and the OD ranged from 0.22 to 0.91 (0.51). In 5-species biofilm, both methods showed no temporal changes (*p* > 0.05) (Fig. 3D). The colonization ranged from 1.80% to 2.68% (0.18), and the OD ranged from 0.32 to 0.74 (0.37).

There were strong positive relationships between microscopy and CV staining in 7-species, 10-species, 15-species, and 20-species biofilms (*R*^2^ ≥ 0.64) (Figs. 4E-4H). Both methods consistently showed that these biofilms exhibited a gradual increase (slope *p* < 0.05) (Figs. 3E-3H). The colonized area ranged from 2.52% to 18.50%(variation coefficient, 0.82), and the OD ranged from 0.24 to 1.34 (0.63) in 7-species biofilm (Fig. 3E). The colonization ranged from 5.18% to 25.10% (0.51), and the OD ranged from 0.30 to 1.41 (0.56) in 10-species biofilm (Fig. 3F). The colonization ranged from 6.10% to 56.14% (0.69), and the OD ranged from 1.04 to 4.77 (0.60) in 15-species biofilm (Fig. 3G). The colonized area ranged from 15.21% to 41.24% (0.36), and the OD ranged from 0.87 to 3.87 (0.51) in 20-species biofilm (Fig. 3H).

### Relationship Between Microscopy and CV Staining

**Fig. 5:**
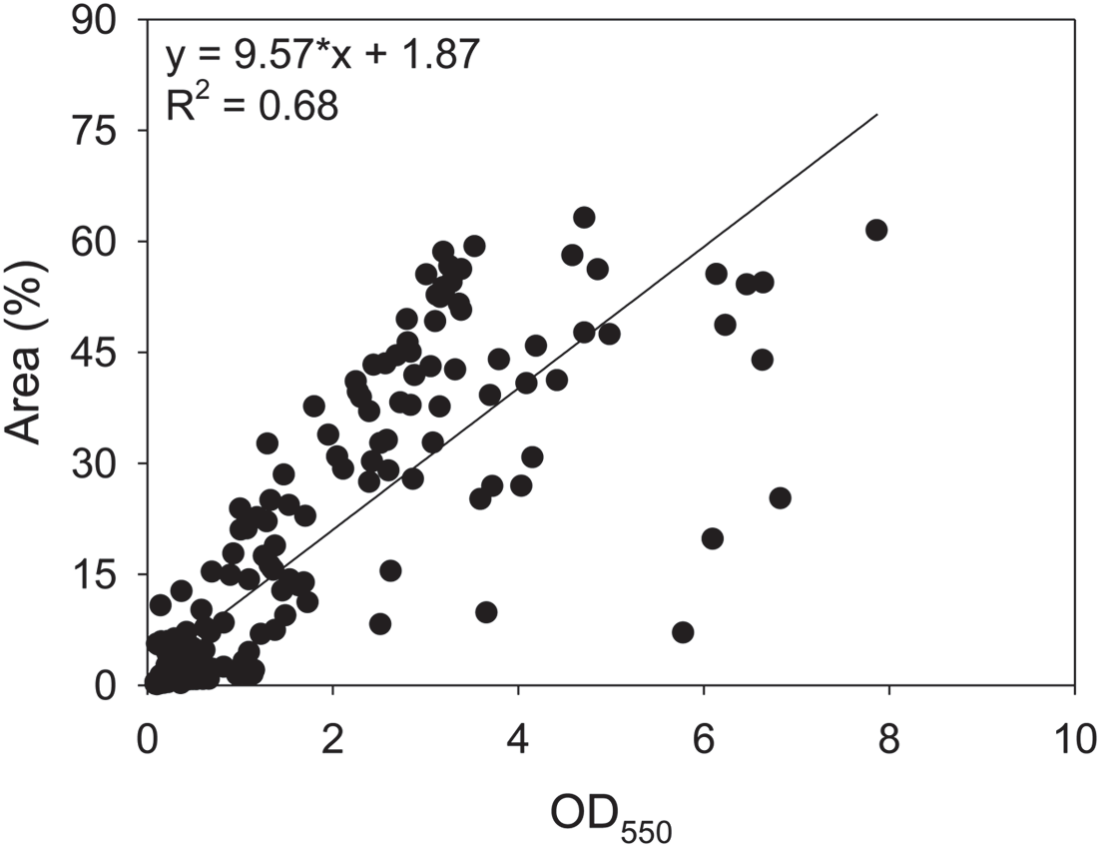
Linearity test between the measurements by epifluorescence microscopy with image analysis and crystal violet staining from all experimental sets in this study. The symbol * indicates statistical significance (*p* < 0.05).

To determine the overall relationship, we performed a linearity test between the microscopy and CV staining measurements from all experimental sets (Fig. 5). The linear regression result exhibited a strong positive relationship (Y = 9.57·*x* + 1.87, *p* < 0.05, *R*^2^ = 0.68). This model indicates that the area colonized by biofilms increased by 9.57% points as the OD increased by 1.

## Discussion

In this study, we evaluated epifluorescence microscopy with image analysis as a quantification method for multispecies biofilms in comparison with CV staining. Microscopy with image analysis is fundamentally different from CV staining for measuring biofilm quantity. Microscopy with image analysis is an absolute quantification method to measure the extent of surface colonization by biofilms, whereas CV staining enables estimation of the total amount of cells and EPS present in biofilms.

To evaluate microscopy as a quantification assay, we generated variations in biofilm quantity by varying species-sequence combinations or acylase treatments. Both techniques consistently showed considerable variation in quantity of the 16 multispecies biofilms with different species-adding sequences (colonized area, 0.05%−56.60%; and OD, 0.11−7.00) (Fig. 1). Ten multispecies biofilms with different acylase treatment episodes also showed variation in quantity (colonized area, 33.82%−55.60%; and OD, 2.14−3.43) (Fig. 2). Linear regression results showed that colonization extents and OD measurements were strongly correlated in both experiments (*R*^2^, 0.79 and 0.69 for the first and second experiments, respectively). Moreover, a qPCR assay targeting 16S rDNA [16] was performed to further validate both methods for the first experiment. We found that the microbial population also agreed well with the colonization extent (*R*^2^, 0.51) and OD measurement (*R*^2^, 0.74) (data not shown). The high level of correspondence indicates that microscopy is comparable to CV staining for multispecies biofilm quantification. This correspondence level is in line with those reported in previous studies that have compared methods for mono-species biofilm quantification [14, 20]. For example, Djordjevic *et al*. [20] reported a correlation between the measurements by CV staining and microscopy (*R*^2^ = 0.66) for quantification of *Listeria monocytogenes* biofilm. Li *et al*. [14] reported that the results of CV staining were highly comparable to those of the metabolic activity assay (*R*^2^ = 0.92) for quantifying biofilms of the yeast *Candida albicans*. Additionally, a variation coefficient was computed to estimate the extent of variability from a set of measurements for each method. Microscopy and CV staining had similar variation coefficients in both experiments, although the variabilities were considerably different between the two experiments (1.35 vs. 1.21 and 0.17 vs. 0.17 for the first and second experiments, respectively). These results indicate that microscopy is comparable to CV staining in identifying quantitative variation in multispecies biofilms. Thus, microscopy with image analysis is suitable for quantifying multispecies biofilms. However, it was found that the relationship between microscopy and staining was discrepant between the first and second experiments (Figs. 1 and 2) (*i.e.*, an area of 50% corresponds to an OD value of 6 in Fig. 1, and 55% corresponds to OD 3 in Fig. 2). Epifluorescence microscopy may be unable to reflect the 3D expansion of biofilms because the surface area colonized by biofilms does not always correspond to the height when biofilms develop [29]. Our observation suggests an important aspect of the microscopic assay that colonized area is not necessarily proportional to biofilm biomass. In addition, microscopy has been shown to be promising for multi- or mono-species biofilm quantifications in this and previous studies. Further comparison for the microscopic quantification of mono- and multi-species biofilms is warranted to elucidate the assay precision, as biofilms vary greatly in structure, size, and composition.

For further comparison between the two methods, temporal changes were observed in eight biofilms (AS and soil consortia plus six synthetic consortia) for 14 days. First, both methods revealed substantial differences in quantity among the biofilms (Fig. 3). Colonization extent did not appear to match the measurement by CV staining in the four biofilms (AS, soil, 3-species, and 5-species consortia), which occupied an area of less than 3.7%(*R*^2^ ≤ 0.25). However, there were strong correlations between the measurements by microscopy and CV staining in the other biofilms (7-, 10-, 15-, and 20-species consortia) that colonized more than 18.7% of the area (*R*^2^ ≥ 0.64). These results indicate that microscopy cannot accurately measure the extent of surface colonization by biofilm below the threshold colonization level. The microscopic quantification method used in this study is for estimating surface colonization of biofilm microcolonies on randomly selected focal areas on the bottom surface, whereas CV staining measures all biofilms present in a well. Biofilm development is a multistage process in which microorganisms attach to a surface, form a biofilm colony, and then mature the biofilm [5, 30]. Biofilm colonies are unlikely to be observed in randomly selected focal areas at an early stage of development. Thus, the major limitation for microscopy with image analysis probably is the tendency to underestimate the quantity of biofilm colonies when they develop on the surface below a certain level. However, there were similar variation coefficients in microscopy and CV staining in the four biofilms in which the two methods were comparable in quantification (0.82 vs. 0.63, 0.51 vs. 0.56, 0.69 vs. 0.60, and 0.36 vs. 0.51 for 7-, 10-, 15-, and 20-species biofilms, respectively). The good agreement on quantity and variation between the two methods indicates that microscopy with image analysis can be an efficient tool for monitoring temporal variations in multispecies biofilms although it is unable to quantify the biofilms below a certain development level.

Linear regression analysis between the measurements by microscopy and CV staining from all experimental sets exhibited a strong positive relationship (Y = 9.57·*x* + 1.87, *p* < 0.05, *R*^2^ = 0.68) (Fig. 5). We extrapolated from the model equation that biofilm saturates the surface when the OD reaches approximately 10.4, because colonization extent increases by 9.6% points with 1 OD. The high correspondence level between the surface coverage and OD measurement confirmed that microscopy with image analysis is a promising method for quantifying multispecies biofilms. There are several inherent advantages and disadvantages to microscopy with image analysis as a quantification method. Most importantly, microscopy can achieve absolute quantification of biofilm colonizing surfaces [19, 31]. Biofilm quantification results can be directly compared between mono-species and multispecies, among species, or among laboratories [31, 32]. Another major advantage of microscopy is its ability to observe spatial and temporal successions of biofilm colonization [33]. It may be possible to achieve non-destructive analysis of biofilm colonization, allowing time-series analysis of biofilm colonization at varied spatial scales in natural or engineering environments, unlike other methods, such as gravimetric analysis, CV staining, qPCR, and metabolic activity assay in which biofilm samples must be destroyed. Moreover, sample preparation and quantification by microscopy can be faster and more cost-effective than that by other methods (*e.g.*, < 1 h vs. > 3 h for single samples by microscopy and CV staining, respectively). However, the microscopic quantification method suggested in this study has several intrinsic biases: the method neglects biofilm thickness in biofilm quantification and assumes that the biofilm is evenly distributed across a surface. These biases can lead to significant errors in quantification when biofilms seldom develop at an early stage (*i.e.*, biofilm colonies are seldom found in randomly selected focal areas), unlike other methods covering all biofilms on surface. Moreover, the method cannot further discern variations in biofilms when biofilms saturate the surface. A concurrent use of microscopy and staining or qPCR assay may be able to overcome the intrinsic limitations raised by this study. Collectively, our findings suggest that epifluorescence microscopy with image analysis is an easy, rapid, and reliable method for the quantification of multispecies biofilms. Specifically, it would be promising to explore the spatial and temporal dynamics of multispecies biofilms.

## Supplemental Materials

Supplementary data for this paper are available on-line only at http://jmb.or.kr.

## Figures and Tables

**Table 1:** Bacterial isolates used in this study.

No.	Isolate	Source	Culture collection	Reference
1	*Acinetobacter* sp. YS01	Wetland soil	KCTC 82588	In this study
2	*Bacillus* sp. AS03	Activated sludge	KCTC 43305	In this study
3	*Escherichia coli*	KCTC^a^	KCTC 2791	In this study
4	*Enterobacter* sp. YS02	Wetland soil	KCTC 82586	In this study
5	*Sphingobium xanthum*	FBCC^b^	FBCC 500366	In this study
6	*Sphingopyxis* sp. NM1	Soil	KCTC 32429	Jeong *et al*. 2014
7	*Microbacterium* sp. NM2	Soil	KCTC 29496	Jeong *et al*. 2018
8	*Staphylococcus warneri*	KCTC^a^	KCTC 3340	In this study
9	*Xanthomonas translucens*	FBCC^b^	FBCC 500042	In this study
10	*Agromyces* sp. FS01	Forest soil	KCTC 49588	Noh *et al*. 2021
11	*Arthrobacter* sp. MF02	Mud flat	KCTC 49587	Noh *et al*. 2021
12	*Burkholderia* sp. FS12	Forest soil	KCTC 82584	Noh *et al*. 2021
13	*Burkholderia* sp. MF09	Mud flat	KCTC 82615	In this study
14	*Novosphingobium* sp. FS10	Forest soil	KCTC 82582	Noh *et al*. 2021
15	*Micrococcus* sp. MF01	Mud flat	KCTC 49590	Noh *et al*. 2021
16	*Mucilaginibacter* sp. FS06	Forest soil	KCTC 82589	Noh *et al*. 2021
17	*Mycolicibacterium* sp. MF04	Mud flat	KCTC 49591	Noh *et al*. 2021
18	*Paraburkholderia* sp. FS13	Forest soil	KCTC 82583	Noh *et al*. 2021
19	*Pedobacter* sp. FS05	Forest soil	KCTC 82590	Noh *et al*. 2021
20	*Pseudomonas* sp. MF13	Mud flat	KCTC 82587	Noh *et al*. 2021
21	*Rhizobium* sp. MF11	Mud flat	KCTC 82581	Noh *et al*. 2021
22	*Rhodobacter* sp. MF12	Mud flat	KCTC 82614	Noh *et al*. 2021
23	*Rhodococcus* sp. FS03	Forest soil	KCTC 49589	Noh *et al*. 2021
24	*Tumebacillus* sp. FS08	Forest soil	KCTC 43304	Noh *et al*. 2021

^a^Korean Collection for Type Cultures (KCTC).

^b^Freshwater Bioresources Culture Collection (FBCC).
