# Supplementary material for: Epifluorescence Microscopy with Image Analysis as a Promising Method for Multispecies Biofilm Quantification
Source: J Microbiol Biotechnol. 2023 Jan 18;33(3):348–55. doi: 10.4014/jmb.2209.09045 (PMC10084751; doi:10.4014/jmb.2209.09045)

J. Microbiol. Biotechnol.
https://doi.org/10.4014/jmb.2209.09045

# Supplementary Tables and Figures

## Epifluorescence Microscopy with Image Analysis as a Promising Method for Multispecies Biofilm Quantification

Ji Won Lee, So-Yeon Jeong* and Tae Gwan Kim*

Department of Microbiology, Pusan National University, Pusan 46241, Republic of Korea

* Corresponding Authors:

So-Yeon Jeong / E-mail address: jeongsy@pusan.ac.kr

Tae Gwan Kim / E-mail address: tkim@pusan.ac.kr

**Table S1. Sequences of bacterial species to be added.**

| No. | Day 1 | Day 2 | Day 3 | Day 4 |
|---|---|---|---|---|
| 1 | A | A | B | B |
| 2 | A | B | B | B |
| 3 | A | S | B | B |
| 4 | A | X | B | B |
| 5 | B | B | B | B |
| 6 | B | A | B | B |
| 7 | B | S | B | B |
| 8 | B | X | B | B |
| 9 | S | S | B | B |
| 10 | S | A | B | B |
| 11 | S | B | B | B |
| 12 | S | X | B | B |
| 13 | X | X | B | B |
| 14 | X | A | B | B |
| 15 | X | B | B | B |
| 16 | X | S | B | B |

A, *Acinetobacter* sp. YS01; B, *Bacillus* sp. AS03; S, *Sphingopyxis* sp. NM1; X, *Xanthomonas translucens*.

**Table S2. Different daily acylase treatment episodes.**

<table>
<tr><th>Treatment</th><th>Day 1</th><th>Day 2</th><th>Day 3</th><th>Day 4</th><th>Day 5</th></tr>
<tr><td>1</td><td colspan="5">No acylase</td></tr>
<tr><td>2</td><td>No acylase</td><td colspan="4">Acylase</td></tr>
<tr><td>3</td><td colspan="2">No acylase</td><td colspan="3">Acylase</td></tr>
<tr><td>4</td><td colspan="3">No acylase</td><td colspan="2">Acylase</td></tr>
<tr><td>5</td><td colspan="4">No acylase</td><td>Acylase</td></tr>
<tr><td>6</td><td colspan="5">Acylase</td></tr>
<tr><td>7</td><td>Acylase</td><td colspan="4">No acylase</td></tr>
<tr><td>8</td><td colspan="2">Acylase</td><td colspan="3">No acylase</td></tr>
<tr><td>9</td><td colspan="3">Acylase</td><td colspan="2">No acylase</td></tr>
<tr><td>10</td><td colspan="4">Acylase</td><td>No acylase</td></tr>
</table>

Acylase, acylase was added to a final concentration of 10 mg $l^{-1}$.
No acylase, acylase was not added.

**Table S3. Bacterial composition of six synthetic consortia.**

| Assemblage | | | | | |
|---|---|---|---|---|---|
| 3 | 5 | 7 | 10 | 15 | 20 |
| 22 | 22 | 22 | 22 | 22 | 22 |
| 24 | 24 | 24 | 24 | 24 | 24 |
| 2 | 2 | 2 | 2 | 2 | 2 |
| | 3 | 3 | 3 | 3 | 3 |
| | 10 | 10 | 10 | 10 | 10 |
| | | 8 | 8 | 8 | 8 |
| | | 14 | 14 | 14 | 14 |
| | | | 21 | 21 | 21 |
| | | | 20 | 20 | 20 |
| | | | 19 | 19 | 19 |
| | | | | 17 | 17 |
| | | | | 13 | 13 |
| | | | | 15 | 15 |
| | | | | 11 | 11 |
| | | | | 18 | 18 |
| | | | | | 12 |
| | | | | | 9 |
| | | | | | 23 |
| | | | | | 16 |
| | | | | | 4 |

2, *Bacillus* sp. AS03; 3, *Escherichia coli*; 4, *Enterobacter* sp. YS02; 8, *Staphylococcus warneri;* 9, *Xanthomonas translucens*; 10, *Agromyces* sp. FS01; 11, *Arthrobacter* sp. MF02; 12, *Burkholderia* sp. FS12; 13, *Burkholderia* sp. MF09; 14, *Novosphingobium* sp. FS10; 15, *Micrococcus* sp. MF01; 16, *Mucilaginibacter* sp. FS06; 17, *Mycolicibacterium* sp. MF04; 18, *Paraburkholderia* sp. FS13; 19, *Pedobacter* sp. FS05; 20, *Pseudomonas* sp. MF13; 21, *Rhizobium* sp. MF11; 22, *Rhodobacter* sp. MF12; 23, *Rhodococcus* sp. FS03; and 24, *Tumebacillus* sp. FS08

**Figure S1. Biofilm micrographs obtained using epifluorescence microscopy (Fig. 1).** Micrographs were obtained from 10 random focal spots for each sample (triplicates per treatment). A micrograph was randomly selected for each treatment. The scale bar represents 200 μm.

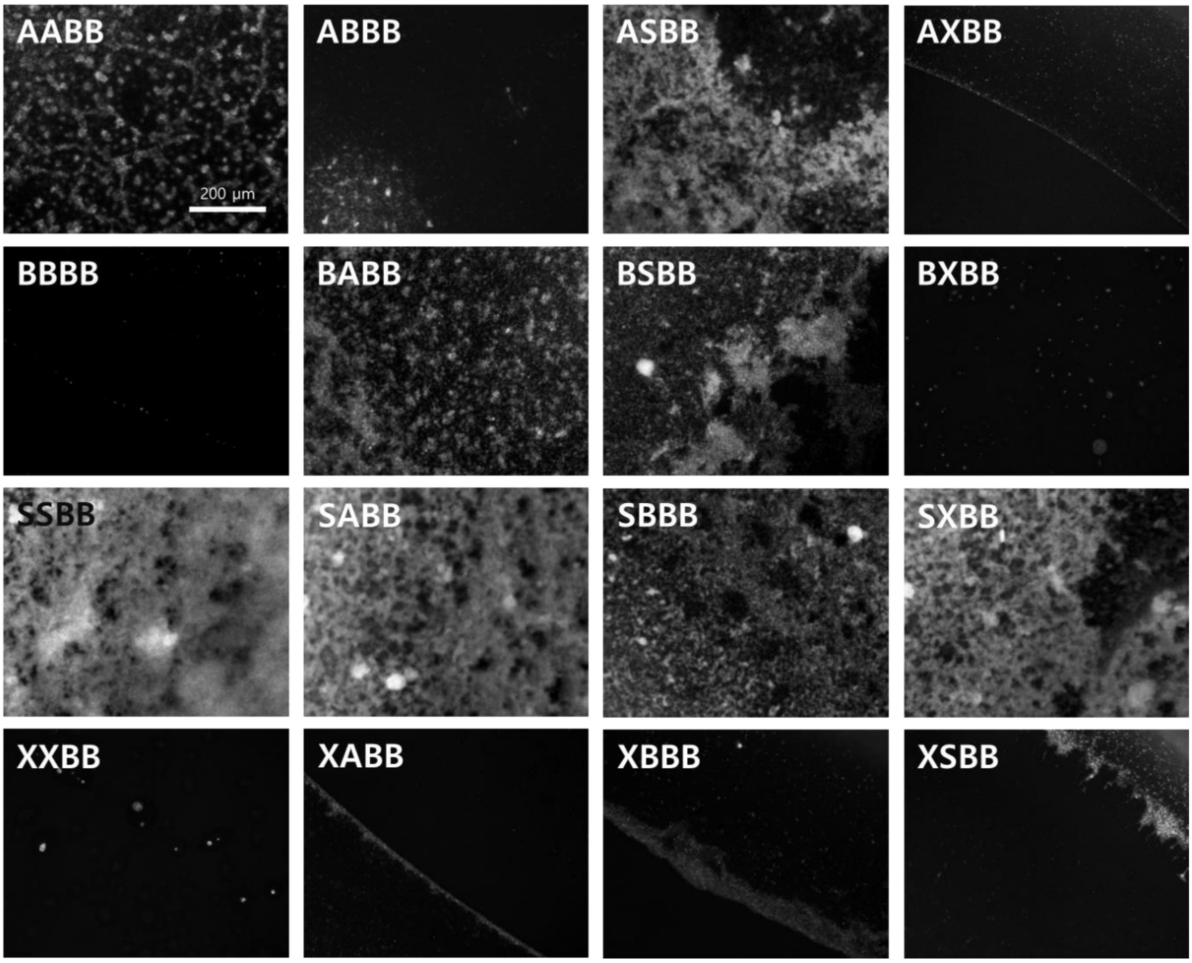

**Figure S2. Biofilm micrographs obtained using epifluorescence microscopy (Fig. 2).** Micrographs were obtained from 10 random focal spots for each sample (triplicates per treatment). A micrograph was randomly selected for each treatment. The scale bar represents 200 µm.

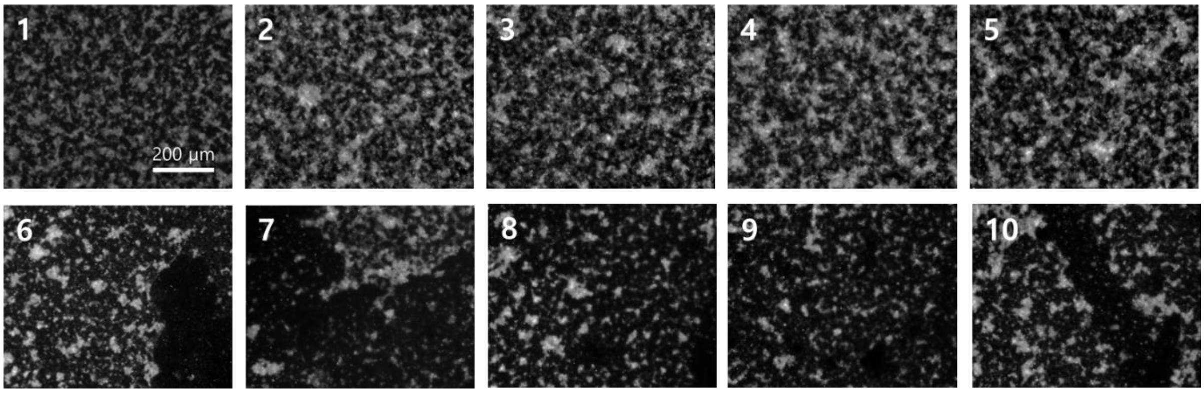

**Figure S3. Biofilm micrographs obtained using epifluorescence microscopy.**

Micrographs were obtained from 10 random focal spots for each sample (triplicates per treatment). A micrograph was randomly selected for each treatment. The scale bar represents 200 µm.

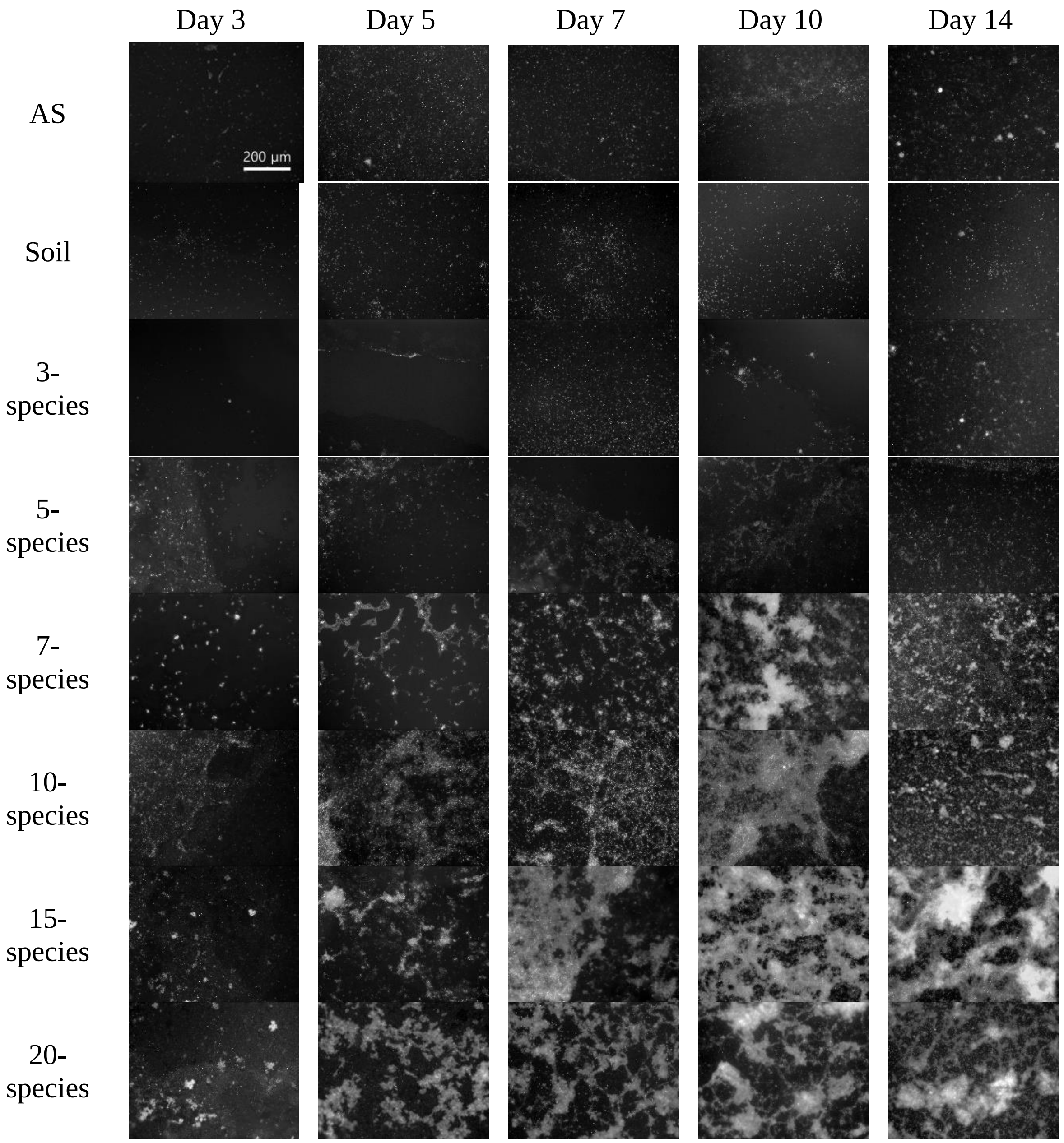

Supplement: Supplementary file 1 [file jmb-33-3-348-supple.pdf]
